# Elevated homocysteine levels predict impaired liver and renal function in women of reproductive age with polycystic ovary syndrome: a *post-hoc* analysis of a randomized controlled trial

**DOI:** 10.3389/fnut.2025.1688690

**Published:** 2026-01-08

**Authors:** Xi Luo, Wang-Yu Cai, Xiao-Ke Wu

**Affiliations:** 1 Department of Gynecology and Obstetrics, Second Affiliated Hospital, Zhejiang Chinese Medical University, Hangzhou, China; 2 Department of Gynecology and Obstetrics, First Affiliated Hospital, Zhejiang Chinese Medical University, Hangzhou, China; 3 Department of Gynecology and Obstetrics, First Affiliated Hospital, Heilongjiang University of Chinese Medicine, Harbin, China

**Keywords:** homocysteine, liver function, renal function, pcos, randomized controlled trial

## Abstract

**Background:**

Polycystic ovary syndrome (PCOS) is associated with liver and kidney injury. Homocysteine (Hcy) may serve as a link between liver/kidney health and PCOS. This research aims to evaluate the relationship between serum Hcy levels and indicators of liver and renal function in women of reproductive age with PCOS.

**Methods:**

A post-hoc analysis of a randomized controlled trial is performed in this study. A total of 1,000 women diagnosed with PCOS were enrolled from secondary and tertiary hospitals in China. At baseline, we collected demographic information and measured metabolic factors, sex hormone levels, liver and renal function parameters, and Hcy levels. Women with an Hcy level >15 μmol/L were diagnosed with hyperhomocysteinemia (HHcy). Parameters associated with liver function were aspartate transferase (AST), alanine transferase (ALT), bilirubin, and total bile acid, while indicators of renal function were blood urea nitrogen (BUN), creatinine (CREA), cystatin C, and β2 microglobulin. Additionally, renal function was assessed using the glomerular filtration rate (eGFR).

**Results:**

Of the 938 PCOS women with available data, 149 (15.9%) were diagnosed with HHcy, while 789 (84.1%) had normal Hcy levels. Compared to the normal Hcy group, the HHcy group exhibited increased levels of AST (12.5 vs. 15.7, *p* < 0.001), ALT (8.7 vs. 10.3, *p* = 0.041), indirect bilirubin (4.2 vs. 4.7, *p* = 0.013), CREA (41.8 vs. 48.7, *p* < 0.001), BUN (4.3 vs. 4.7, *p* < 0.001), β2 microglobulin (1.3 vs. 1.5, *p* < 0.001), and cystatin C (0.4 vs. 0.6, *p* < 0.001) levels, along with a lower eGFR (120.9 vs. 127.7, *p* < 0.001). The levels of AST, ALT, CREA, and BUN were found to be significantly increased in all Hcy quartiles, while eGFR was decreased across Hcy quartiles (*P* for trend <0.05). After adjusting for BMI, waist circumference, glucose, TC, and HDL, Hcy levels were positively associated with AST (*β* = 0.268, *p* < 0.001), total bilirubin (*β* = 0.127, *p* < 0.001), direct bilirubin (*β* = 0.038, *p* = 0.002), indirect bilirubin (*β* = 0.087, *p* < 0.001), total bile acid (*β* = 0.051, *p* = 0.015), CREA (*β* = 0.812, *p* < 0.001), BUN (*β* = 0.051, *p* < 0.001), β2 microglobulin (*β* = 0.026, *p* < 0.001), cystatin C (*β* = 0.013, *p* < 0.001), and eGFR (*β* = −0.810, *p* < 0.001); however, there was no association betweenHcy levels and ALT (*β* = 0.051, *p* = 0.381) after adjusting for confounding factors.

**Conclusion:**

Elevated Hcy levels are significantly associated with indicators of impaired liver and renal function in women of reproductive age with PCOS.

## Introduction

Polycystic ovary syndrome (PCOS) is one of the most prevalent endocrine and reproductive disorders that affect women during their reproductive years. PCOS typically manifests as oligomenorrhea, hyperandrogenism, and polycystic ovaries (1) and accounts for almost half of anovulatory infertility in reproductive-aged women. As women age, reproductive abnormalities may improve and endocrinological disturbances can gradually emerge, leading to conditions such as insulin resistance, obesity, and dyslipidemia (2).

Previous research has suggested associations between PCOS and liver and kidney dysfunction. Both the kidney and liver are involved in the maintenance of homeostasis in the body. Despite their distinct functions, these organs are closely related in various physiological and pathological processes. The kidney is mainly responsible for filtering blood, removing waste, and regulating the body’s fluid balance, while the primary functions of the liver include metabolite detoxification and protein synthesis. In the general population, non-alcoholic fatty liver disease (NAFLD), which manifests as excessive lipid deposition in the liver, is one of the most common liver diseases (3). Chronic kidney disease (CKD), associated with a progressive decline in kidney function, is one of the most prevalent forms of kidney disease (4). PCOS is closely linked to NAFLD and, more broadly, to fatty liver disease (FLD) (5–7), while a positive causal link was observed between PCOS and CKD (8). These associations may be partly explained by certain characteristics of PCOS, such as hyperandrogenism, obesity, insulin resistance, and dyslipidemia; however, the underlying mechanisms are not fully understood.

Homocysteine (Hcy) is an amino acid that is produced during the metabolic conversion of methionine to cysteine. It is usually considered a marker for cardiovascular disease. Elevated levels of homocysteine, or hyperhomocysteinemia (HHcy), are recognized as an independent cardiovascular risk factor (9–12). Individuals with Hcy levels exceeding 10 μmol/L have a markedly greater risk of all-cause mortality, with every additional 1 μmol/L rise in Hcy corresponding to a 2% increase in cardiovascular death (13). PCOS is independently linked to elevated Hcy levels (14, 15), regardless of obesity, insulin resistance, or androgen concentrations (16). Epidemiological studies have indicated that HHcy is associated with both liver and renal diseases (17–19). Combined with the above evidence, this suggests that Hcy dysregulation may explain the association between liver/renal injury and PCOS. Few studies have investigated the associations between Hcy levels and liver/renal function in women with PCOS. The objective of the present study is to explore a potential independent association between serum Hcy levels and parameters of liver/renal function in women with PCOS.

## Materials and methods

### Study design and population

This study presents a post-hoc analysis of data from a previously published randomized controlled trial (20). PCOS was diagnosed using the modified Rotterdam criteria (21), which require the presence of oligomenorrhea (menstrual interval ≥35 days, or ≤ 8 menses/year) or amenorrhea. In addition, one or both of the following criteria must be met: hyperandrogenism (Ferriman-Gallwey score ≥5 or total testosterone ≥1.67 nmol/L) and the presence of polycystic ovaries (> 12 follicles or ovarian volume >10 mL on ultrasonography).

### Data measurement

At the baseline visit, all participants underwent a standardized physical examination conducted by trained staff. Trained staff measured height, weight, waist circumference, systolic blood pressure (SBP), and diastolic blood pressure (DBP), and then calculated body mass index (BMI). At baseline, fasting blood samples were drawn from every participant on the third day of their menstrual cycle in the follicular phase. Blood samples were stored at −20 °C and transported to the central laboratory at Heilongjiang University of Chinese Medicine (ISO15189 certified) for analysis. HHcy was defined as >15 μmol/L based on the cutoff value of the laboratory. The measurements of sex hormones included serum levels of estradiol, luteinizing hormone (LH), follicle-stimulating hormone (FSH), total testosterone (TT), and sex hormone-binding globulin (SHBG). The metabolic parameters were glucose, insulin, high-density lipoprotein (HDL), low-density lipoprotein (LDL), triglycerides (TG), and total cholesterol (TC). Homeostatic model assessment-insulin resistance (HOMA-IR) was calculated from the glucose and insulin measurements. The parameters of liver function were aspartate transferase (AST), alanine transferase (ALT), total bilirubin, direct bilirubin, indirect bilirubin, and total bile acid. The indicators of renal function were blood urea nitrogen (BUN), creatinine (CREA), cystatin C, and beta 2 microglobulin. The estimated glomerular filtration rate (eGFR) was also calculated from CREA to assess renal function using the Chronic Kidney Disease Epidemiology Collaboration (CKD-EPI) formula (22).

### Laboratory assessments

Hcy levels were measured using the enzymatic method (Gcell, Beijing, China). Plasma glucose was quantified using the hexokinase method (Maccura, Chengdu, China). Estradiol, LH, FSH, TT, and fasting insulin were analyzed using electrochemiluminescence immunoassays (Roche Diagnostics, Basel, Switzerland). SHBG was measured using a chemiluminescence immunoassay (Siemens, Munich, Germany). The lipid panel was measured by the direct-method assay method (Wako Diagnostics, Osaka, Japan). Liver enzymes were measured using the International Federation of Clinical Chemistry method (23, 24). Bilirubin was measured using the vanadate oxidation method (Wako Diagnostics, Osaka, Japan). Total bile acid was measured using the enzyme cycle method (Maccura, Chengdu, China). BUN was measured using the ultraviolet-glutamate dehydrogenase method (Maccura, Chengdu, China), CREA was measured using the sarcosine oxidase method (Maccura, Chengdu, China), cystatin C levels were determined using the latex immunoturbidimetric method (Gcell, Beijing, China), and β2 microglobulin was measured using the immunoturbidimetric method (Maccura, Chengdu, China).

### Statistical analysis

This study is a post-hoc analysis of data from a previously published randomized controlled trial (Clinicaltrials.gov, identifier: NCT01573858). All statistical analyses were conducted using SPSS version 24.0 (IBM Corp., Armonk, NY, USA). Only women with complete data were included in this current study. Normally distributed data are presented as mean ± standard deviation, and non-normally distributed data are presented as median with interquartile ranges. Group comparisons were performed using independent *t*-tests and the Mann–Whitney U-test. Trends across Hcy quartiles were assessed using linear regression. The association between Hcy and liver/renal function parameters was examined for linear regression assumptions, including normality and constant variance, and when linear regression assumptions were met, we used linear regression models. Multivariate linear regression models evaluated associations between Hcy levels and liver/renal function parameters, adjusting for potential confounders. A two-sided *p*-value of <0.05 was considered statistically significant.

## Results

A total of 1,000 women were enrolled in the original randomized controlled trial, among whom 938 had complete data on Hcy levels and indicators of liver and renal function, and these 938 women were included in this current post-hoc analysis. Table 1 summarizes the baseline characteristics of the study participants. The mean age and BMI of the participants were 27.9 ± 3.3 years and 24.2 ± 4.3 kg/m^2^, respectively. Of the 938 women with PCOS, 149 (15.9%) were diagnosed with HHcy, while 789 (84.1%) had normal Hcy levels. Compared to the normal Hcy group, the BMI (23.9 vs. 25.4, *p* < 0.001) and waist circumference (84.5 vs. 88.8, *p* < 0.001) were higher in the HHcy group, while the glucose (5.1 vs. 4.7, *p* < 0.001), TC (4.8 vs. 4.6, *p* = 0.035), and HDL (1.3 vs. 1.2, *p* < 0.001) levels were lower in the HHcy group. In terms of liver and renal function parameters, the HHcy group had significantly higher levels of AST (12.5 vs. 15.7, *p* < 0.001), ALT (8.7 vs. 10.3, *p* = 0.041), indirect bilirubin (4.2 vs. 4.7, *p* = 0.013), CREA (41.8 vs. 48.7, *p* < 0.001), BUN (4.3 vs. 4.7, *p* < 0.001), β2 microglobulin (1.3 vs. 1.5, *p* < 0.001), and cystatin C (0.4 vs. 0.6, *p* < 0.001), and lower eGFR values (120.9 vs. 127.7, *p* < 0.001). The associations between renal function parameters and Hcy levels were thus stronger.

**Table 1:** Characteristics of all women and women with normal Hcy and HHcy.

Variables	All (*N* = 938)	Normal Hcy (*N* = 789)	HHcy (*N* = 149)	*p*-value
Age	27.9 ± 3.3	27.9 ± 3.4	27.7 ± 3.1	0.487
BMI (kg/m^2^)	24.2 ± 4.3	23.9 ± 4.2	25.4 ± 4.2	**<0.001**
Waist circumference (cm)	85.4 ± 11.5	84.5 ± 11.3	88.8 ± 11.7	**<0.001**
SBP (mmHg)	110 (108–120)	110 (106.5–120)	112 (110–120)	0.072
DBP (mmHg)	75 (70–80)	75 (70–80)	75 (70–80)	0.263
Hcy (μmol/L)	8.3 ± 4.9	6.8 ± 2.8	16.6 ± 5.2	**<0.001**
Sex hormone				
LH (mIU/ml)	9.4 (6.1–14.2)	9.4 (6.1–14.3)	9.4 (6.4–13.7)	0.827
FSH (mIU/ml)	6.0 (5.0–7.1)	6.0 (5.1–7.1)	5.9 (5.0–7.0)	0.553
E2 (pmol/L)	199.5 (158.9–265.8)	198.5 (155.9–264.4)	201.6 (170.7–267.1)	0.216
TT (nmol/L)	1.6 (1.2–2.0)	1.6 (1.2–2.1)	1.6 (1.4–2.0)	0.073
SHBG (nmol/L)	33.7 (21.7–54.8)	33.4 (22.1–55.2)	31.1 (19.8–51.9)	0.188
Metabolic				
Glucose (mmol/L)	5.0 ± 1.0	5.1 ± 0.9	4.7 ± 1.2	**<0.001**
Insulin (pmol/L)	98.1 ± 108.5	97.6 ± 111.8	95.7 ± 72.4	0.834
HOMA-IR	3.4 ± 5.5	3.5 ± 5.9	2.9 ± 2.3	0.289
TG (mmol/L)	1.6 ± 0.9	1.6 ± 0.9	1.5 ± 0.9	0.347
TC (mmol/L)	4.7 ± 1.1	4.8 ± 1.1	4.6 ± 1.0	**0.035**
HDL (mmol/L)	1.3 ± 0.4	1.3 ± 0.4	1.2 ± 0.3	**0.001**
LDL (mmol/L)	3.0 ± 0.9	3.0 ± 0.9	2.9 ± 0.8	0.070
Liver function				
AST (U/L)	13.0 ± 7.3	12.5 ± 6.9	15.7 ± 9.0	**<0.001**
ALT (U/L)	9.0 ± 8.6	8.7 ± 8.3	10.3 ± 9.8	**0.041**
Total Bilirubin (μmol/L)	6.4 ± 3.2	6.3 ± 3.2	6.8 ± 3.1	0.104
Direct Bilirubin (μmol/L)	2.1 ± 1.7	2.2 ± 1.6	2.1 ± 2.3	0.669
Indirect Bilirubin (μmol/L)	4.3 ± 2.3	4.2 ± 2.3	4.7 ± 2.6	**0.013**
Total bile acid	2.0 ± 2.9	1.9 ± 2.4	2.2 ± 4.8	0.210
Renal function				
CREA (μmol/L)	42.9 ± 10.8	41.8 ± 10.5	48.7 ± 10.4	**<0.001**
BUN (mmol/L)	4.4 ± 1.3	4.3 ± 1.3	4.7 ± 1.0	**<0.001**
Beta 2 Microglobulin (mg/L)	1.3 ± 0.4	1.3 ± 0.4	1.5 ± 0.4	**<0.001**
Cystatin C (mg/L)	0.46 ± 0.15	0.4 ± 0.1	0.6 ± 0.2	**<0.001**
eGFR (ml/min per 1.73 m^2^)	126.6 ± 12.5	127.7 ± 12.0	120.9 ± 13.7	**<0.001**

All data were presented with mean ± standard deviation or median (interquartile range). Independent t-test was used to compare the means between groups.

Homocysteine (Hcy), hyperhomocysteinemia (HHcy), body mass index (BMI), systolic blood pressure (SBP), diastolic blood pressure (DBP), luteinizing hormone (LH), follicle-stimulating hormone (FSH), estradiol (E2), total testosterone (TT), sex hormone-binding globulin (SHBG), homeostatic model assessment-insulin resistance (HOMA-IR), triglycerides (TG), total cholesterol (TC), high-density lipoprotein (HDL), low-density (LDL), aspartate transferase (AST), alanine transferase (ALT), blood urea nitrogen (BUN), creatinine (CREA), and estimated glomerular filtration rates (eGFR). Bold values indicated statistical significances.

**Figure 1:**
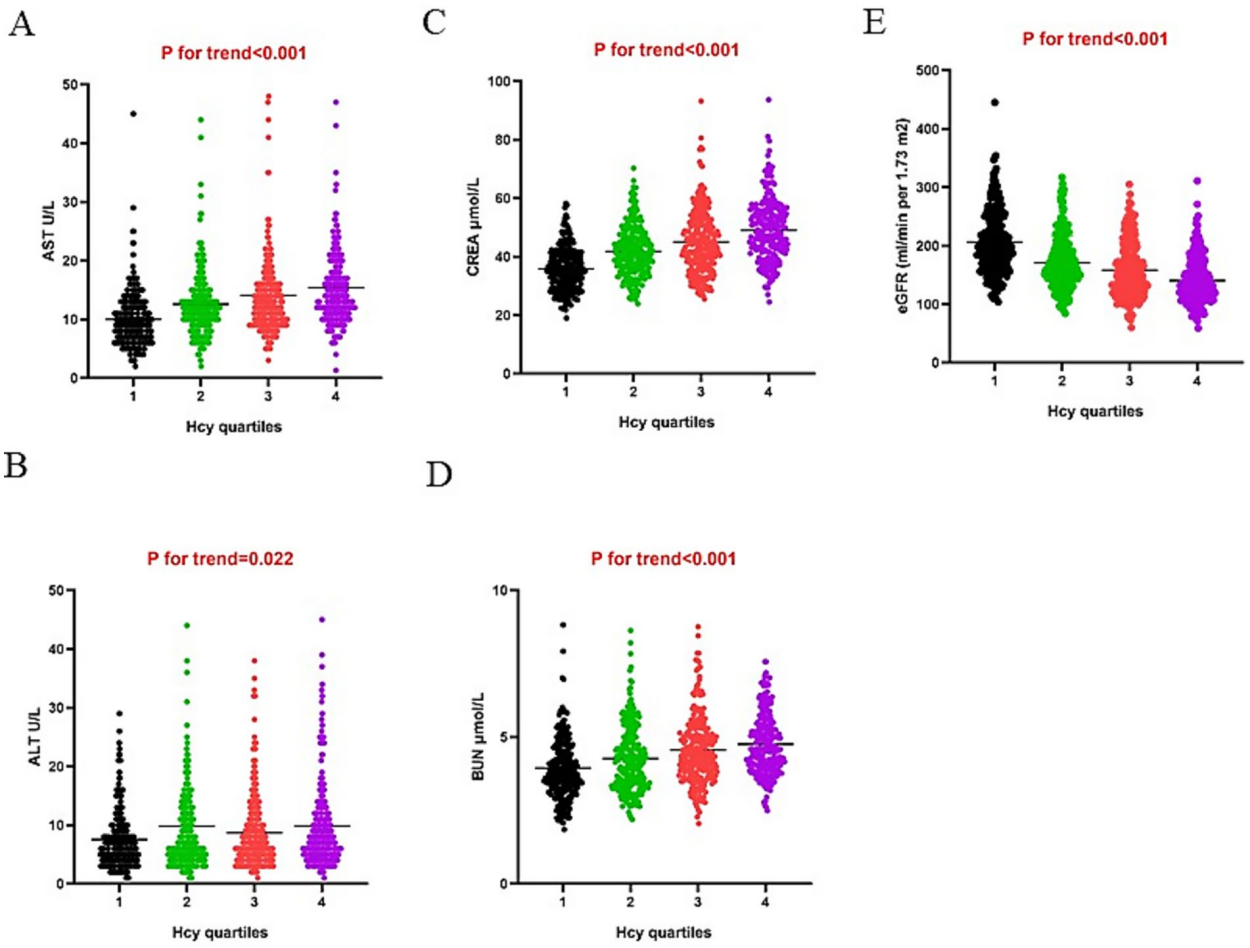
**(A)** AST, **(B)** ALT, **(C)** CREA, **(E)** BUN, and **(E)** eGFR across Hcy quartiles.

Figure 1 shows the values of liver and renal function indicators as quartiles of Hcy. The ranges of the Hcy quartiles were Q1 (0–5.01), Q2 (5.01–7.55), Q3 (7.55–10.465), and Q4 (>10.465). The levels of AST, ALT, CREA, and BUN were significantly increased, while eGFR values were decreased across the Hcy quartiles (*p* < 0.001). The relationship was strong between the renal function parameters and Hcy.

Table 2 presents unadjusted linear regression results assessing the relationship between Hcy and liver and renal function. Hcy levels were significantly positively associated with AST (*β* = 0.329, *p* < 0.001), total bilirubin (*β* = 0.111, *p* < 0.001), direct bilirubin (*β* = 0.026, *p* = 0.026), indirect bilirubin (*β* = 0.084, *p* < 0.001), total bile acid (*β* = 0.045, *p* = 0.024), CREA (*β* = 0.839, *p* < 0.001), BUN (*β* = 0.052, *p* < 0.001), β2 microglobulin (*β* = 0.030, *p* < 0.001), and cystatin C (*β* = 0.014, *p* < 0.001), and was marginally associated with ALT (*β* = 0.113, *p* = 0.051). Hcy was negatively associated with eGFR (*β* = −0.840, *p* < 0.001).

**Table 2:** Linear association between Hcy (independent) and liver and renal function parameters (dependent) in univariate linear regression.

Variables	Coefficients with 95% CI	*p*-value
AST (U/L)	0.329 (0.234 to 0.424)	**<0.001**
ALT (U/L)	0.113 (−0.001 to 0.227)	0.051
Total Bilirubin (μmol/L)	0.111 (0.070 to 0.153)	**<0.001**
Direct Bilirubin (μmol/L)	0.026 (0.003 to 0.049)	**0.026**
Indirect Bilirubin (μmol/L)	0.084 (0.053 to 0.115)	**<0.001**
Total bile acid	0.045 (0.006 to 0.084)	**0.024**
CREA (μmol/L)	0.839 (0.707 to 0.972)	**<0.001**
BUN (mmol/L)	0.052 (0.036 to 0.069)	**<0.001**
Beta 2 Microglobulin (mg/L)	0.030 (0.026 to 0.035)	**<0.001**
Cystatin C (mg/L)	0.014 (0.013 to 0.016)	**<0.001**
eGFR (ml/min per 1.73 m^2^)	−0.840 (−0.998 to −0.682)	**<0.001**

Linear regression analyses were used to examine the correlation between Hcy and liver/renal function parameters.

Aspartate transferase (AST), alanine transferase (ALT), blood urea nitrogen (BUN), creatinine (CREA), and estimated glomerular filtration rates (eGFR). Bold values indicated statistical significances.

**Figure 2:**
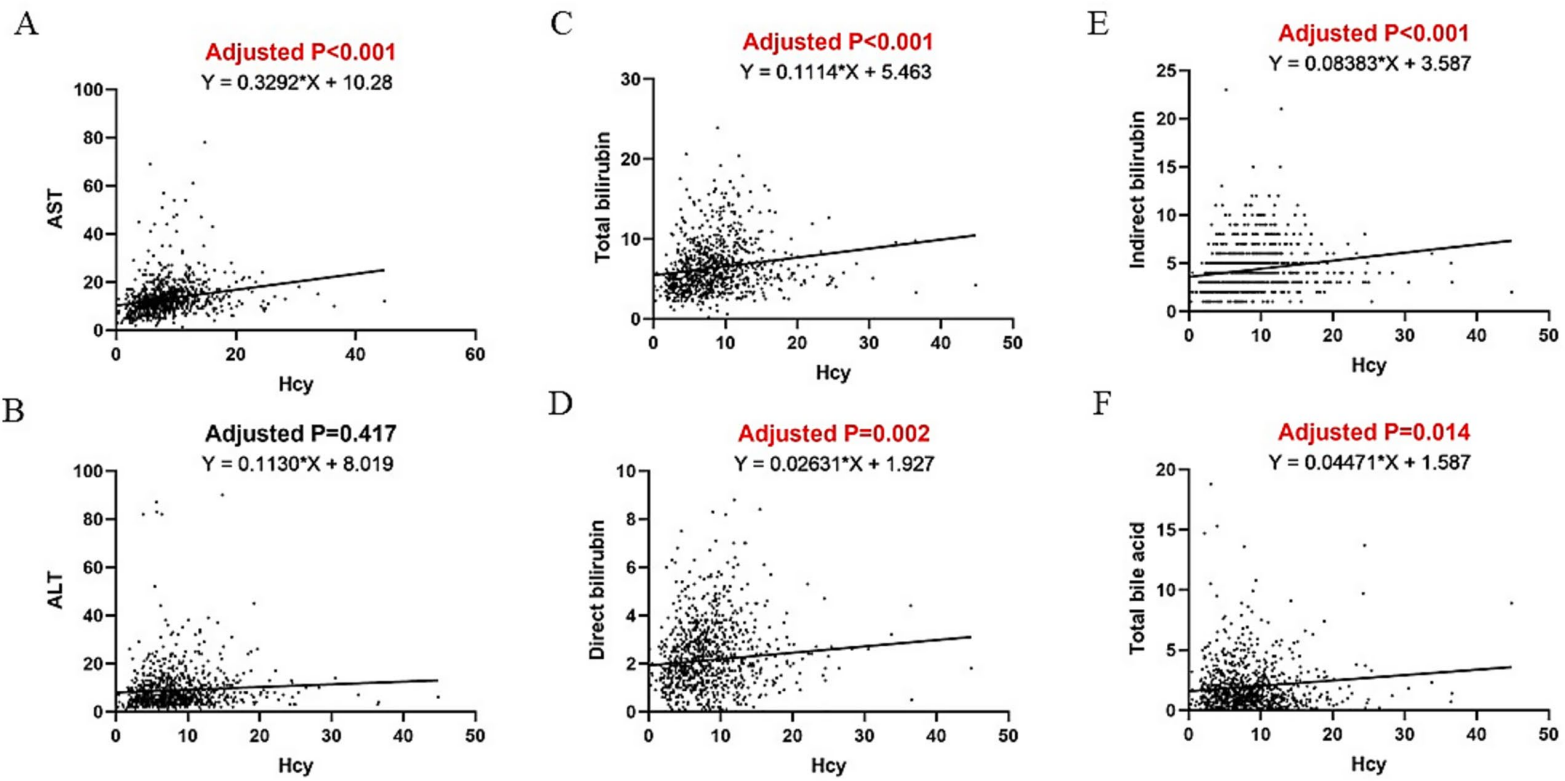
Linear association between Hcy and **(A)** AST, **(B)** ALT, **(C)** total bilirubin, **(D)** direct bilirubin, **(E)** indirect bilirubin, and **(F)** total bile acid.

**Figure 3:**
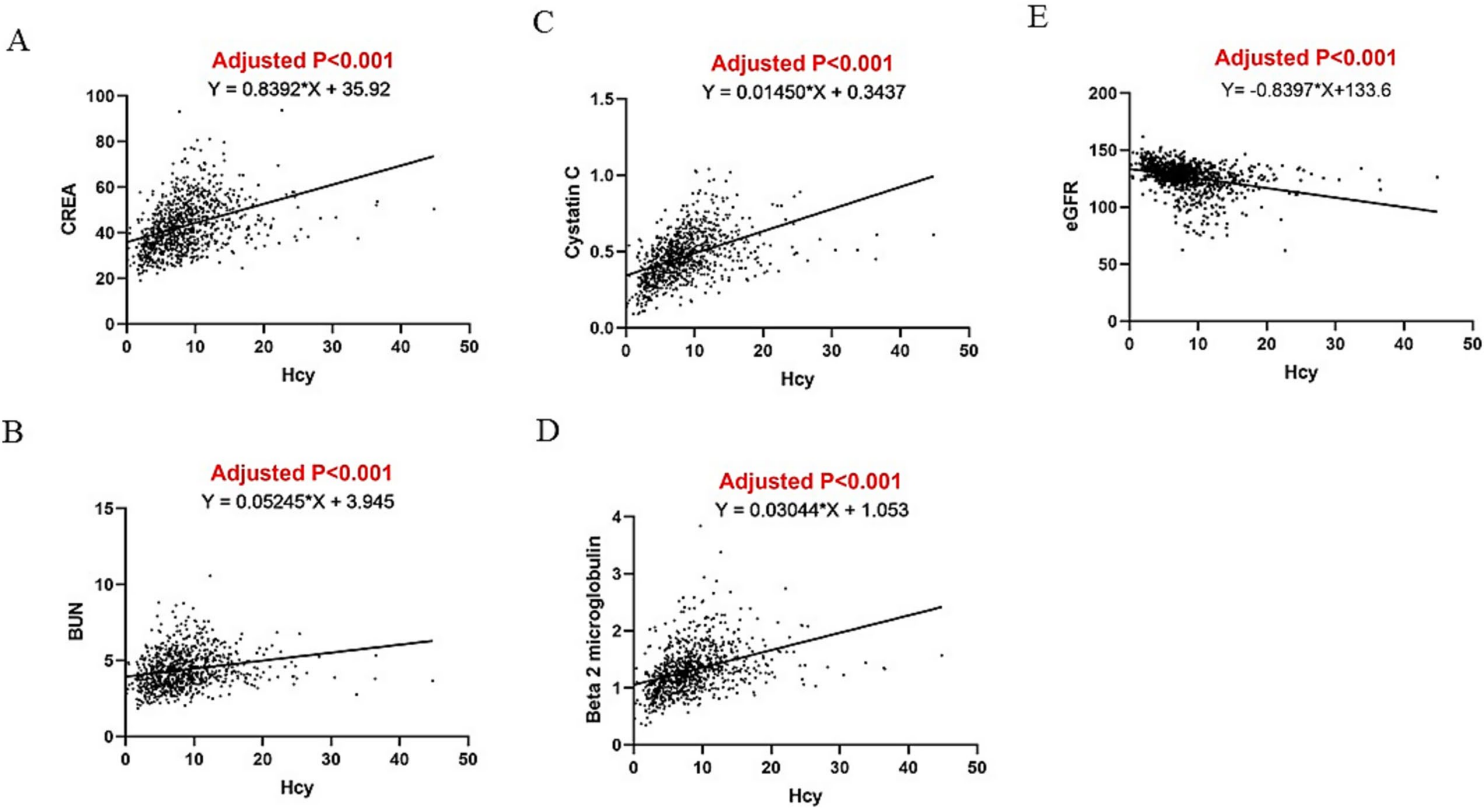
Linear association between Hcy and **(A)** CREA, **(B)** BUN, **(C)** beta 2 microglobulin, **(D)** cystatin **(C)**, and **(E)** eGFR.

Table 3 summarizes the findings of a linear regression analysis that link Hcy levels with liver and renal function parameters after adjusting for BMI, waist circumference, glucose, TC, and HDL. Multivariate linear regression revealed that Hcy levels were positively associated with AST (*β* = 0.268, *p* < 0.001), total bilirubin (*β* = 0.127, *p* < 0.001), direct bilirubin (*β* = 0.038, *p* = 0.002), indirect bilirubin (*β* = 0.087, *p* < 0.001), total bile acid (*β* = 0.051, *p* = 0.015), CREA (*β* = 0.812, *p* < 0.001), BUN (*β* = 0.051, *p* < 0.001), β2 microglobulin (*β* = 0.026, *p* < 0.001), and cystatin C (*β* = 0.013, *p* < 0.001), but not with ALT (*β* = 0.051, *p* = 0.381), after adjusting for confounding factors. Hcy levels were found to be independently negatively associated with eGFR (*β* = −0.810, *p* < 0.001) (Table 3). Figures 2, 3 illustrate the linear associations between Hcy levels and liver/renal function parameters and adjusted *p*-values.

**Table 3:** Linear association between Hcy (independent) and liver and renal function parameters (dependent) in multivariate linear regression.

Variables	Coefficients with 95% CI*	*p*-value
AST (U/L)	0.268 (0.170 to 0.366)	**<0.001**
ALT (U/L)	0.051 (−0.063 to 0.166)	0.381
Total Bilirubin (μmol/L)	0.127 (0.084 to 0.169)	**<0.001**
Direct Bilirubin (μmol/L)	0.038 (0.015 to 0.062)	**0.002**
Indirect Bilirubin (μmol/L)	0.087 (0.055 to 0.119)	**<0.001**
Total bile acid	0.051 (0.010 to 0.091)	**0.015**
CREA (μmol/L)	0.812 (0.674 to 0.951)	**<0.001**
BUN (mmol/L)	0.051 (0.034 to 0.069)	**<0.001**
Beta 2 Microglobulin (mg/L)	0.026 (0.021 to 0.031)	**<0.001**
Cystatin C (mg/L)	0.013 (0.011 to 0.015)	**<0.001**
eGFR (ml/min per 1.73 m^2^)	−0.810 (−0.975 to −0.644)	**<0.001**

*Linear regression analyses were used to examine the correlation between Hcy and liver/renal function parameters after adjusting for BMI, waist circumference, glucose, TC, and HDL.

Aspartate transferase (AST), alanine transferase (ALT), blood urea nitrogen (BUN), creatinine (CREA), and estimated glomerular filtration rates (eGFR). Bold values indicated statistical significances.

## Discussion

Serum Hcy is routinely monitored in clinical settings as a measure of cardiovascular health. In this study of women of reproductive age with PCOS, Hcy levels were observed to be negatively associated with indicators of liver and renal function, irrespective of BMI, waist circumference, glucose, TC, and HDL. The results suggest that higher serum Hcy levels in women with PCOS are predictive of an increased risk of developing liver and kidney diseases in the future.

Several studies have linked PCOS with impaired liver and kidney function. One study found that increased urinary albumin excretion and decreased glomerular filtration rates were more frequent in women with PCOS, both of which are markers for renal damage (25). Women with PCOS may have normal renal function, but they still have higher urinary albumin excretion and serum uric acid levels than those in the controls (26). Animal studies have indicated that PCOS model rats had increased numbers of CD4 + CD28^null^ T cells in the kidney, which suggests potential renal injury (27). In terms of liver function, a study has found increased ALT levels (>35 U/L) in 30% of PCOS patients attending a fertility clinic (28). During pregnancy, PCOS is associated with a higher risk of pregnancy-induced hypertension, including gestational hypertension, preeclampsia/eclampsia, or hemolysis, elevated liver enzymes, and low platelet count (HELLP) syndrome (29, 30). A proposed hepato-ovarian axis suggests that NAFLD may exacerbate hepatic and systemic insulin resistance that may play important roles in the pathophysiology of PCOS (31). To link PCOS with liver/renal health, the most likely mechanisms are insulin resistance and hyperadrogenism. Insulin resistance and high testosterone levels may cause elevated liver lipogenesis and impair liver function. As for renal function, insulin resistance and high testosterone levels may activate oxidative stress and local inflammation, leading to damage in the glomerulus.

There have been only a few studies examining the putative association between Hcy levels and liver/renal function. The present results suggest that these associations involve different manifestations of increased Hcy levels. On the one hand, as Hcy is produced and catabolized in the liver and kidneys, damage to these organs may lead to increased Hcy levels. On the other hand, an elevated Hcy level may accelerate the progression of liver and renal damage. The liver and kidneys are both important metabolic organs and are responsible for the metabolism of nutrients, endotoxins, and waste (32). The onset and progression of liver and kidney injury are usually accompanied by oxidative stress (33, 34). The kidney is a highly vascular organ, and the complex and intricate organization of the renal microcirculation is essential to renal function. Vascular dysfunction typically leads to abnormalities in kidney function (35). Hcy is considered to cause oxidative stress and result in vascular endothelial dysfunction (36, 37). HHcy impairs the integrity of the vessel walls and, in turn, affects the vascular tone by reducing the levels of nitric oxide (NO), leading to inflammation, oxidative stress, and cytokine release, and consequent adverse effects on endothelial function (38). These effects of Hcy on the promotion of oxidative stress and vascular dysfunction may be the mechanism underlying the adverse effects of Hcy on liver and renal function, indicating that the presence of HHcy might be an early warning of liver and renal function impairment.

An additional interesting observation is the positive link between Hcy and bile acid concentrations in women with PCOS. A previous study suggested a potential axis involving the gut microbiota, bile acid metabolism, and interleukin-22, and PCOS pathology (39). Additionally, another study reported elevations in both primary and conjugated bile acids in PCOS, suggesting that bile acids may play a pivotal role in the development of PCOS (40). Hcy exhibits cytotoxic effects and can induce oxidative stress, inflammatory responses, and endothelial dysfunction in liver cells, thereby inhibiting the expression of key enzymes involved in bile acid synthesis. Dysregulation of bile acid synthesis is involved in the regulation of glucose and lipid metabolism and therefore exacerbates insulin resistance. Our findings indicate that dysregulated Hcy and bile acid metabolism may jointly underpin the pathogenesis of PCOS.

This study investigated the relationship between serum Hcy levels and indicators of liver and kidney function in a large cohort of women with PCOS. This study has several strengths. First, the study population was from a well-designed, large sample, prospective, randomized controlled trial. Second, all the participants had a comprehensive evaluation of liver and kidney function parameters in a central laboratory. However, the study has several limitations. First, it was a post-hoc analysis of a published randomized controlled trial; therefore, the sample size was not calculated for this study, which possibly weakens the statistical power. Second, information on Hcy levels was missing for some participants, which may also reduce the statistical power. Furthermore, this study enrolled only Chinese Han women. Further prospective multicenter studies are necessary to verify the findings in different populations.

## Conclusion

The study findings provide evidence for associations between Hcy levels and liver/kidney health in women with PCOS. Overall, higher levels of serum Hcy were associated with those of indicators of impaired liver and kidney function in women with PCOS. Future prospective investigations that explore the effects of Hcy-improving interventions on liver and renal health in PCOS women are needed to further the understanding of the role of Hcy.

## Data Availability

The original contributions presented in the study are included in the article/supplementary material, further inquiries can be directed to the corresponding author/s.
